# Prenatal Diagnosis of Interrupted Inferior Vena Cava with Azygos Continuation: A Case Report

**DOI:** 10.3390/reports8040266

**Published:** 2025-12-14

**Authors:** Martina Billeci, Gianfranco Morreale, Ferdinando Antonio Gulino, Francesco Giuseppe Cannone

**Affiliations:** 1 Department of Gynaecology and Obstetrics, Lentini Hospital, Contrada Colle Roggio, SS194, 96016 Lentini, Italy; martinabilleci31@gmail.com (M.B.); gianfrancomorreale13@gmail.com (G.M.); francesco.cannone@hotmail.it (F.G.C.); 2 Unit of Gynecology and Obstetric, Department of Human Pathology of Adults and Developmental Age, University Hospital “G. Martino”, 98100 Messina, Italy

**Keywords:** interrupted inferior vena cava, azygos continuation, prenatal diagnosis, fetal echocardiography, congenital vascular anomaly

## Abstract

Inferior vena cava (IVC) disruption with continuation of the azygos is a rare congenital vascular abnormality that can be detected prenatally via high-resolution ultrasound. We present a case of isolated discontinuation of IVC, diagnosed during a routine abnormal scan of the second trimester, confirmed by fetal echocardiography, with an uneventful neonatal outcome. In accordance with the literature, we discuss the diagnostic approach, clinical significance and long-term implications of this vascular variant. We want to emphasize the importance of recognizing this anomaly and differentiating isolated cases from those associated with other congenital malformations.

## 1. Introduction and Clinical Significance

Interrupted inferior vena cava (IVC) with azygos continuation is an uncommon vascular anomaly that may present in isolation or as part of complex congenital syndromes. It can be detected prenatally and has important implications for diagnosis, counseling, and postnatal care. In this article, we present a case of isolated IVC interruption diagnosed prenatally, followed by a review of the literature to provide a broader understanding of this vascular anomaly.

## 2. Case Presentation

**Figure 1:**
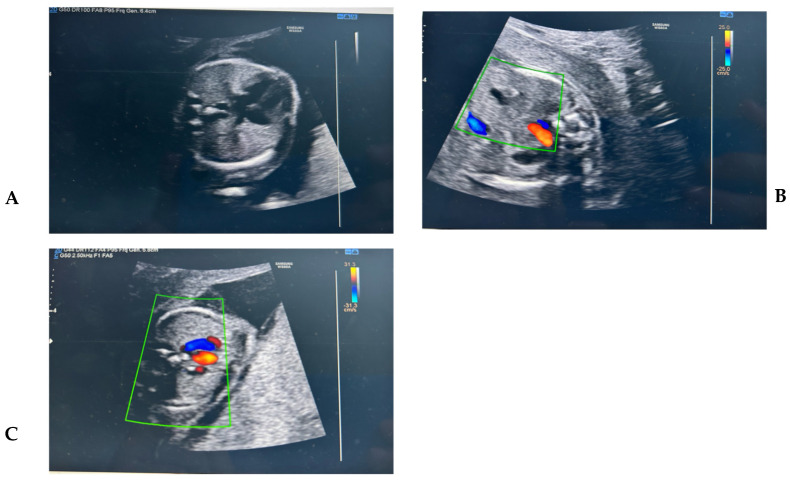
(**A**) The four-chamber view shows a dilated azygos vein (Az) located posterior to the descending aorta (Ao), a finding known as the “double vessel sign.” (**B**) The normal vascular conformation at this level typically includes the aorta (Ao) located near the spine on the left, a small and usually non-visualized azygos vein, and the inferior vena cava (IVC) positioned anterior and to the right of the aorta. (**C**) The three-vessel and trachea (3VT) view shows a dilated azygos vein (Az) draining into the superior vena cava (SVC). This finding reflects a compensatory mechanism in cases of interrupted inferior vena cava (IVC) with azygos continuation.

A 35-year-old woman, gravida 2 para 1, presented for a second-trimester morphological ultrasound at 20 weeks of gestation. She had previously delivered vaginally after a pregnancy with a normal karyotype confirmed by chorionic villus sampling. During that first pregnancy, she had undergone an invasive procedure due to an increased nuchal translucency detected at the first-trimester scan. The current pregnancy had been uneventful, with normal non-invasive prenatal testing and a reassuring first-trimester ultrasound. During the anomaly scan, an abnormal venous return was identified. A vessel was noted posterior to the descending aorta in the four-chamber view, consistent with the “double vessel sign.” Further examination in the bicaval view revealed absence of the intrahepatic segment of the IVC, and a dilated superior vena cava (SVC) was evident in the three-vessel view. A thinner vessel was traced from the infrahepatic IVC to the SVC, indicating azygos continuation. The patient was referred to a tertiary center for fetal echocardiography, which confirmed the diagnosis. Genetic counseling was offered, but the patient declined invasive testing and opted to continue the pregnancy. A male infant weighing 2.8 kg was delivered vaginally at 38 weeks of gestation. The neonatal course was uneventful. Postnatal echocardiography performed at 6 weeks confirmed the prenatal diagnosis of isolated IVC interruption with azygos continuation. No cardiac or extracardiac abnormalities were found. The infant was scheduled for routine follow-up due to the theoretical risk of arrhythmias or venous insufficiency in later life. (Figure 1A) The four-chamber view shows a dilated azygos vein (Az) located posterior to the descending aorta (Ao), a finding known as the “double vessel sign”. (Figure 1B)The normal vascular conformation at this level typically includes the aorta (Ao) located near the spine on the left, a small and usually non-visualized azygos vein, and the inferior vena cava (IVC) positioned anterior and to the right of the aorta. (Figure 1C) The three-vessel and trachea (3VT) view shows a dilated azygos vein (Az) draining into the superior vena cava (SVC). This finding reflects a compensatory mechanism in cases of interrupted inferior vena cava (IVC) with azygos continuation.

## 3. Discussion

Interrupted inferior vena cava with azygos or hemiazygos continuation results from abnormal embryologic development of the cardinal venous system. While it may occur as an isolated vascular variant, it is more frequently associated with complex cardiac and extracardiac anomalies. In particular, the anomaly represents an important imaging marker of suspected heterotaxy syndrome, especially left isomerism. Berg et al. described how left atrial isomerism is commonly associated with interruption of the IVC, atrioventricular septal defects, rhythm disturbances, and abnormalities in the position of abdominal organs, including polysplenia or asplenia. Therefore, whenever azygos or hemiazygos continuation is identified, a systematic evaluation of situs, cardiac anatomy, venous connections, spleen number and morphology, and abdominal organ arrangement is recommended.

Another important aspect is the potential clinical impact on feto–maternal Doppler surveillance. Although isolated IVC interruption is usually benign, alterations in systemic venous return may influence venous Doppler interpretation in fetuses who are also small for gestational age. Recent evidence by Lobmaier et al. highlights the importance of longitudinal venous Doppler assessment in the surveillance of late-onset SGA fetuses; in this context, awareness of variant venous anatomy can prevent misinterpretation of ductus venosus or hepatic venous flow patterns.

Advances in first-trimester imaging have also improved early detection of systemic venous anomalies. According to von Kaisenberg et al., detailed assessment of fetal thoracic vessels between 11 and 13 + 6 weeks—including the ductus venosus, systemic venous return, and abdominal situs—allows earlier suspicion of IVC interruption, particularly when the nuchal translucency is increased or other subtle markers of heterotaxy are present. Although most diagnoses still occur in the second trimester, the increasing resolution of early fetal anatomical scans supports the role of first-trimester evaluation when feasible [1,2,3,4,5,6,7,8].

In the present case, the anomaly was isolated, and both prenatal and postnatal examinations excluded heterotaxy, cardiac malformations, and extracardiac anomalies. This aligns with previous studies reporting an excellent prognosis when azygos continuation is the sole finding. Nevertheless, long-term follow-up is advisable due to the theoretical risk of arrhythmias and potential venous insufficiency later in life.

## 4. Conclusions

Interrupted inferior vena cava with azygos continuation is a rare but important prenatal finding. Accurate diagnosis using ultrasound is crucial for guiding counseling and perinatal care. In isolated cases, the prognosis is generally favorable. Our report supports the role of detailed fetal imaging in detecting this condition and highlights the importance of a multidisciplinary approach to prenatal counseling.

## Data Availability

The original contributions presented in this work are included in the article. Further inquiries can be directed to the corresponding author.
